# Two-year quality of life after robot-assisted radical prostatectomy according to pentafecta criteria and cancer of the prostate risk assessment (CAPRA-S)

**DOI:** 10.1038/s41598-021-04289-2

**Published:** 2022-01-07

**Authors:** Theodoros Karagiotis, Jorn H. Witt, Thomas Jankowski, Mikolaj Mendrek, Christian Wagner, Andreas Schuette, Nikolaos Liakos, Pawel Rachubinski, Katarina Urbanova, Matthias Oelke, Mykyta Kachanov, Sami-Ramzi Leyh-Bannurah

**Affiliations:** 1 Prostate Center Northwest, Department of Urology, Paediatric Urology and Urooncology, St. Antonius-Hospital, Gronau, Germany; 2 Martini-Klinik Prostate Cancer Center Hamburg-Eppendorf, Hamburg, Germany

**Keywords:** Urological cancer, Prostate cancer, Quality of life

## Abstract

The quality of life (QoL) of men with optimal outcomes after robot-assisted radical prostatectomy (RARP) is largely unexplored. Thus we assessed meaningful changes of QoL measured with the EORTC QLQ-C30 24 months after RARP according to postsurgical Cancer of the Prostate Risk Assessment score (CAPRA-S) and pentafecta criteria. 2871 prostate cancer (PCa) patients with completed EORTC QLQ-C30 were stratified according to CAPRA-S, pentafecta (erectile function recovery, urinary continence recovery, biochemical-recurrence-free survival (BFS), negative surgical margins) and 90-day Clavien–Dindo-complications (CDC) ≤ 3a. Multivariable logistic regression analyses (LRM) aimed to predict improvement of EORTC QoL. Mean preoperative QoL values did not significantly differ between CAPRA-S low- (LR) vs. high-risk (HR, 75.7 vs. 75.2; p = 0.7) and pentafecta vs. non-pentafecta groups (75.6 vs. 75.2; p = 0.6). After RARP, stable QoL rates for CAPRA-S LR vs. HR and pentafecta were 30, 26 and 30%, respectively. Corresponding improved QoL rates were 44, 32 and 47%. In LRM, CAPRA-S and pentafecta criteria were independent predictors of improved QoL. We conclude that most favourable combined outcomes after RARP might confer stable or even improved QoL but up to one third of patients might experience deterioration. This warrants further investigation how to capture the underlying cause and to address and potentially solve these perceived negative effects despite successful RARP.

## Introduction

Radical prostatectomy (RP) remains a standard treatment option for localised prostate cancer (PCa) with well documented local and long-term PCa control^1–3^. RP patients ideally wish to recover quickly and to preserve a satisfactory long-term quality of life (QoL). Moreover, compared to open RP, robot-assisted radical prostatectomy (RARP) might confer added benefit of better perioperative outcomes^4–6^. Monitoring these outcomes and associated QoL is essential to improve preoperative patient counselling, RARP technique and post-RARP rehabilitation^7,8^.

It is highly conceivable but largely unexplored that certain combinations of favourable pathological, functional or oncological outcomes of RARP are perceived with best QoL. Specifically, the pentafecta criteria includes a combination of biochemical recurrence (BCR)-free survival, recovery of urinary continence and of erectile function at a specific time-point, as well as negative surgical margin status and absence of surgical complications^9,10^. Another favourable combined outcome is the postsurgical Cancer of the Prostate Risk Assessment score low risk score (CAPRA-S)^11^. A low-risk CAPRA-S is based on favourable surgical and pathological characteristics, such as low pre-surgical PSA < 20 ng/ml, pathological Gleason pattern ≤ 4 + 4 and ideally negative surgical margin and localized disease (i.e. no extracapsular extension, seminal vesicle invasion and lymph node invasion^12^).

For purpose of QoL assessment, the European Organisation for Research and Treatment of Cancer Quality of Life Questionnaire Core 30 (EORTC QLQ-C30) is widely used in cancer patients, including PCa. It is a validated patient-reported outcome measurement (PROM) tool that covers functional scales, symptoms, single items and global QoL^13–15^. Accordingly, it is regularly utilized in clinical trials^16^ that compare treatment methods, e.g. RP vs. radiotherapy^1,17–19^. In daily clinical practice, as in our institution, it also serves for monitoring QoL after RARP over time^20^.

It is of note that previous series on EORTC QLQ-C30 and RP mainly relied on exploratory analyses, e.g. mean change of individual EORTC QLQ-C30 scales over time and did not stratify according to specific functional or oncological scenarios. Moreover, most series omitted multivariable analyses^19,21–24^ and relied on ORP patients^19,21,22^. In general, comprehensive data on RARP and EORTC QLQ-C30 is sparse. To date, none applied the EORTC QLQ-C30 Summary Score in the context of RARP, which incorporate most functional and symptom scales and single items. None applied minimal clinically important differences (MCIDs) after RARP to enable intuitive interpretation of QoL effects. Finally, none dedicatedly examined effect of specific favourable patient-centric combined outcomes such as pentafecta or low-risk CAPRA-S on QoL.

Thus, we examined the impact of CAPRA-S risk categories and pentafecta criteria on EORTC QLQ-C30 global QoL and Summary Score in patients treated with RARP and report relevant respective improvement vs. deterioration rates.

## Material and methods

Overall, 2871 consecutive PCa patients with complete pathological, surgical and complications data who received RARP at our institution between 5/2006 and 12/2018 were identified. Analyses were restricted to men with complete data on EORTC QLQ C30 at baseline and 24 months after surgery, who were preoperatively continent and who had corresponding follow-up information of urinary continence recovery and information on baseline erectile function. Patients with neoadjuvant androgen deprivation therapy, suspected metastases or with previous local therapy of the prostate were excluded. RARP specimen grading was performed according to 2014 International Society of Urological Pathology standards^25^.

### EORTC-QLQ-C30 and minimum clinically important differences

The questionnaire includes 30 items that can be summarized into 15 scales, i.e. five functional scales (physical, role, emotional, cognitive and social), three symptom scales (fatigue, nausea/vomiting and pain), six singe items (dyspnea, insomnia, appetite loss, constipation, diarrhea, financial impact of disease) and a global QoL (QL).

For our study we utilized the QL and the EORTC QLQ-C30 Summary Score. Latter is calculated by the sum of 13 scales (QL and financial impact are excluded), divided by the number 13^11,26–28^. For calculation, the symptom scales are reversed to obtain a uniform direction of every scale.

We classified MCIDs, i.e. difference of pre- vs. 24 months postoperative EORTC QLQ-C30 scale, as “stable/no change” based on ≤ 5, “minor” based on > 5–10, “moderate” on > 10–20 and “major” on > 20 points^29^. A positive vs. negative change was classified as improvement vs. deterioration, respectively.

### Outcomes

The CAPRA-S is a scoring system based on pre-surgical PSA, pathological Gleason score, surgical margin status, extracapsular extension, seminal vesicle invasion and lymph node invasion^12,30^. The Patients were stratified according to CAPRA-S into low- (LR), intermediate- (IR) and high-risk (HR) group, based on respective score ranges of 0–2, 3–5 and ≥ 6^12^.

Preoperative potency and recovery of erectile function were defined as combination of International Index of Erectile Function (IIEF-5) score ≥ 18^31^ and/or score ≥ 3 at the second question^32^. Recovery of urinary continence was defined as fulfilling following criteria: up to one pad usage within 24 h (safety pad) or score of ≤ 2 at the International Consultation on Incontinence Questionnaire-Urinary Incontinence Short Form Questions 1 and 2 (“How often do you leak urine?” and “How much urine do you usually leak (whether you wear protection or not)?”)^33^ or finally, the International Continence Society male questionnaire score of ≤ 1 at each of the three questions I2, I3 and I4. For preoperative continence, no preoperative pad usage was allowed. BCR was defined as consecutive PSA rise ≥ 0.2 ng/ml.

Those with recovery of erectile function- and urinary continence and BCR-free-survival within 24 months were classified as having met the trifecta criteria, i.e. ideal functional and oncological outcomes 24 months post-RARP. Those men with preoperative erectile dysfunction, were still modeled as having met the trifecta-criteria if urinary continence recovery and BCR-free-survival within 24 months was observed. Complications were coded according to the Clavien-Dindo classification (CDC)^34^. Finally, those, who met the combined criteria of negative surgical margins, CDC ≤ 3a and 24-months trifecta were classified as pentafecta group (vs. non-pentafecta).

### Statistical analyses

Chi-square test was used for categorical and t-test for continuous variables. For multivariable analyses, we performed logistic regression analyses (LRM) to predict (a) improvement (i.e. at least > 5 points positive change at the individual EORTC QLQ-C30 scale) and (b) absence of deterioration (i.e. stable or even improved EORTC QLQ-C30 scales). LRM was modeled with preoperative EORTC QLQ-C30 scale of interest (QL or Summary Score), 90-day CDC (0-3a[REF] vs. ≥ 3b, i.e. requiring intervention under general anesthesia), CAPRA-S risk groups (LR [REF.] vs. IR vs. HR), which incorporates the surgical margin status, and the 24 months trifecta criteria (non-fulfillment [REF] vs. fulfillment).

All tests were two-sided with a statistical significance set at *p* < 0.05. Analyses were performed with the statistical package for *R* (R foundation for Statistical Computing, version 3.2.2).

### Ethics approval

The institutional review board at the St. Antonius-Hospital, Gronau, approved the retrospective study design and access to the patients’ medical records. All methods were carried out in accordance with the Declaration of Helsinki. Written informed consent was obtained from individual participants in the study.

### Compliance with ethical standards

All authors of this research paper have directly participated in the planning, execution, or analysis of the study. All authors of this paper have read and approved the final version submitted. The contents of this manuscript have not been copyrighted or published previously. The contents of this manuscript are not under consideration for publication elsewhere.

## Results

Table 1 demonstrates the baseline characteristics. Median age of the total cohort was 63 years (IQR 59–67) and median PSA was 7.3 ng/ml (IQR 5.4–10.7). RARP ISUP Gleason Grades 1, 2, 3 and ≥ 4 were 36, 29, 22 and 13%, respectively. Respective proportions of pT2, extracapsular extension, seminal vesicle invasion, positive surgical margin and lymph node invasion were 65, 25, 7.7%, 11 and 6.5%. Based on aforementioned clinicopathological metrics, patients were classified as CAPRA-S low-, intermediate and high-risk in 53, 32 and 15% respectively.

**Table 1:** Baseline characteristics of 2871 prostate cancer patients treated with robot-assisted radical prostatectomy at the Prostate Cancer Center Northwest, Gronau, Germany between 2006 and 2018 stratified according to the CAPRA-S risk group and fulfillment the pentafecta criteria.

Value	All Patients (n2871)		CAPRA-S low risk group (n = 1514)		CAPRA-S intermediate risk group (n = 925)		CAPRA-S high risk group (n = 432)		*p*-value	Pentafecta group (n1469)		Non-pentafecta group (n = 1402)		*p*-value
Age (years), median (IQR)	63	(59–67)	62	(58–66)	64	(60–67)	64	(60–67)	< *0.001*^a^	63	(58–66)	63	(59–67)	*0.002*
Preoperative PSA (ng/ml), median (IQR)7.3	7.3	(5.4–10.7)	5.9	(4.6–7.9)	8.5	(6.4–11.8)	13.8	(10.1–23.3)	< *0.001*^a^	6.7	(5.0–9.4)	8	(5.8–12.0)	< *0.001*
**Pathological ISUP Grade, n (%)**														
1	1033	36%	966	64%	66	7.1%	1	0.2%	< *0.001*^a^	644	44%	389	28%	< *0.001*
2	822	29%	454	30%	325	35%	43	10%		434	30%	388	28%	
3	634	22%	94	6.2%	401	43%	139	32%		278	19%	356	25%	
≥ 4	382	13%	0	0%	133	14%	249	58%		113	7.7%	269	19%	
**Pathological tumor stage, n (%)**														
pT2	1871	65%	1442	95%	416	45%	13	3.0%	< *0.001*^a^	1119	76%	752	54%	< *0.001*
pT3a	719	25%	70	4.6%	441	48%	208	48%		273	19%	446	32%	
pT3b	220	7.7%	2	0.1%	62	6.7%	156	36%		73	5.0%	147	10%	
pT4	61	2.1%	0	0%	6	0.7%	55	13%		4	0.3%	57	4.1%	
Lymph node invasion, n (%)	186	6.5%	0	0%	31	3.4%	155	36%	< *0.001*^a^	48	3.3%	138	10%	< *0.001*
Positive surgical margins, n (%)	326	11%	18	1.2%	123	13%	185	43%	< *0.001*^a^	0	0.0%	326	23%	< *0.001*
**90 days complications according to the Clavien–Dindo classification, n (%)**														
≤ 3a	2802	97.6%	1481	97.8%	901	97.4%	420	97.2%	0.6^a^	1469	100%	1333	95%	< *0.001*
≥ 3b	69	2.4%	33	2.2%	24	2.6%	12	2.8%		0	0%	69	4.9%	
Biochemical recurrence free survival at 24 months after surgery, n (%)	2463	86%	1421	94%	757	82%	285	66%	< *0.001*^a^	1469	100%	994	71%	< *0.001*
Urinary continence recovery at 24 months after surgery, n (%)	2759	96%	1458	96%	894	97%	407	94%	1^a^	1469	100%	1290	92%	< *0.001*
Erectile function recovery at 24 months after surgery, n (%)	1818	63%	1043	69%	545	59%	230	53%	< *0.001*^a^	1469	100%	349	25%	< *0.001*

Significant values are in italics.

*CAPRA-S* the postsurgical Cancer of the Prostate Risk Assessment score, *ISUP* International Society of Urological Pathology.

^a^CAPRA-S low risk vs. CAPRA-S high risk.

The 90-day CDC rates ≤ 3a of the total cohort were 97.6%. At 24 months post-RARP, rates of erectile function and urinary continence recovery, as well as BCR-free survival of the total cohort were 63, 96 and 86%, respectively. Based on negative surgical margin and aforementioned metrics, 24-month pentafecta rates were 51%.

Table 2 demonstrates pre- vs. post-operative mean QL and Summary Score and respective MCID proportions, stratified according to CAPRA-S risk groups and pentafecta vs. non-pentafecta groups. Before RARP there was no statistically significant difference in the QL (75.7, SD 19.4 vs. 75.2, SD 19.4; p = 0.7) nor in Summary Score (91.0, SD 9.2 vs. 91.2 SD 9.2; p = 0.8) between CAPRA-S LR vs. HR. Conversely, 24 months after RARP, there was a statistically significant difference in the QL (79.3, SD 19.3 vs. 73.7, SD 20.7; p < 0.001), as well as Summary Score (90.2, SD 11.5 vs. 87.6, SD 13.4; p < 0.001).

**Table 2:** EORTC QLQ-C30 characteristics of 2871 prostate cancer patients treated with robot-assisted radical prostatectomy at the Prostate Cancer Center Northwest, Gronau, Germany between 2006 and 2018 stratified according to the CAPRA-S risk group and fulfillment the pentafecta criteria.

Value	All patients (n = 2871)		CAPRA-S low risk group (n = 1514)		CAPRA-S intermediate risk group (n = 925)		CAPRA-S high risk group (n = 432)		*p*-value	Pentafecta group (n = 1469)		Nonpentafecta group (n = 1402)		*p*-value
Preoperative Global Quality of Life (QL), mean (SD)	75.4	19.2	75.7	19.4	75.1	18.9	75.2	19.4	0.7^a^	75.6	19.7	75.2	18.7	0.6
Preoperative EORTC QLQ-C30 Summary Score, mean (SD)	91.0	9.1	91.0	9.2	90.9	8.9	91.2	9.2	0.8^a^	91.3	9.0	90.7	9.3	0.08
Global Quality of Life (QL) at 24 months after surgery, mean (SD)	77.5	19.5	79.3	19.3	76.5	19.1	73.7	20.7	< *0.001*^a^	80.8	18.3	74.1	20.2	< *0.001*
EORTC QLQ-C30 Summary Score at 24 months after surgery, mean (SD)	89.3	11.9	90.2	11.5	88.8	11.6	87.6	13.4	< *0.001*^a^	91.2	10.3	87.4	13.1	< *0.001*
**Global Quality of Life (QL) change at 24 months after surgery, n (%)**														
Major improvement	378	13%	207	14%	115	12%	56	13%	< *0.001*^a^	224	15%	154	11%	< *0.001*
Moderate improvement	441	15%	259	17%	130	14%	52	12%		257	17%	184	13%	
Minor improvement	342	12%	199	13%	112	12%	31	7.2%		206	14%	136	10%	
Stable	834	29%	451	30%	271	29%	112	26%		442	30%	392	28%	
Minor deterioration	274	10%	126	8.3%	94	10%	54	13%		111	7.6%	163	12%	
Moderate deterioration	327	11%	147	10%	114	12%	66	15%		140	10%	187	13%	
Major deterioration	275	10%	125	8.3%	89	10%	61	14%		89	6.1%	186	13%	
**EORTC QLQ-C30 Summary Score change at 24 months after surgery, n (%)**														
Major improvement	41	1.4%	22	1.5%	10	1.1%	9	2.1%	< *0.001*^a^	25	1.7%	16	1.1%	< *0.001*
Moderate improvement	167	5.8%	98	6.5%	47	5.1%	22	5.1%		93	6.3%	74	5.3%	
Minor improvement	307	11%	193	13%	82	8.9%	32	7.4%		181	12%	126	9.0%	
Stable	1579	55%	848	56%	510	55%	221	51%		877	60%	702	50%	
Minor deterioration	350	12%	163	11%	127	14%	60	14%		140	10%	210	15%	
Moderate deterioration	293	10%	136	9.0%	103	11%	54	13%		112	7.6%	181	13%	
Major deterioration	134	4.7%	54	3.6%	46	5.0%	34	7.9%		41	2.8%	93	6.6%	

Significant values are in italics.

*CAPRA-S* the postsurgical Cancer of the Prostate Risk Assessment score, *EORTC QLQ-C30* European Organisation for Research and Treatment of Cancer Quality of Life Questionnaire Core 30, *SD* standard deviation, *QoL* quality of life.

^a^CAPRA-S low risk vs. CAPRA-S high risk.

Before RARP, between pentafecta vs. non-pentafecta groups, there was no statistically significant difference in the QL (75.6, SD 19.7 vs. 75.2, SD 18.7; p = 0.6) nor Summary Score (91.3, SD 9.0 vs. 90.7, SD 9.3; p = 0.08). Conversely, 24 months after RARP, there was a statistically significant difference in the QL (80.8, SD 18.3 vs. 74.1, SD 20.2; p < 0.001), as well as Summary Score (91.2, SD 10.3 vs. 87.4, SD 13.1; p < 0.001).

At 24 months after RARP, between CAPRA-S LR vs. HR, QL remained stable in 30 vs. 26% and improved in 44 vs. 32% (p < 0.001). These amounted to 74 vs. 58% without any deterioration in QL. Similarly, the Summary Score remained stable in 56 vs. 51% and improved in 21 vs. 15% (p < 0.001). These amounted to 77 vs. 66% without any deterioration in the Summary Score.

At 24 months after RARP, between pentafecta vs. non-pentafecta groups, QL remained stable in 30 vs. 28% and improved in 47 vs. 34% (p < 0.001). These amounted to 78 vs. 62% without any deterioration in QL. Similarly, the Summary Score remained stable in 60 vs. 50% and improved in 20 vs. 15% (p < 0.001). These amounted to 80 vs. 65% without any deterioration in the Summary Score.

Table 3 shows the multivariable logistic regression model for prediction of improvement of QL and Summary Score at 24 months after robot-assisted radical prostatectomy**.** LRM included CAPRA-S risk groups and the remaining criteria (i.e. trifecta and CDC) that are utilized for the pentafecta criteria, as well as the preoperative mean score of either QL or Summary Score. Specifically, LRM for prediction of improved QL at 24 months after RARP, yielded following independent predictors: higher preoperative score (OR 0.94, 95% CI 0.93–0.94; p < 0.001), higher risk group of CAPRA-S (HR vs. LR[REF] OR 0.60, 95% CI 0.46–0.78; p < 0.001) as well as 90-d surgical complications that require surgical intervention in general anesthesia (CDC ≥ 3b vs. 0-3a[REF] OR 0.49, 95% CI 0.24–0.99; p = 0.048) were associated with lower chance of improved QoL. Conversely, fulfilment of trifecta criteria was associated with virtually doubled odds of improved QL (OR 1.91, 95% CI 1.59–2.29; p < 0.001).

**Table 3:** Multi- and univariable logistic regression model for prediction of improvement of Global Quality of Life (QL) and EORTC QLQ-C30 Summary Score at 24 months after robot-assisted radical prostatectomy.

Value	Global quality of life (QL) improvement			EORTC QLQ-C30 Summary Score improvement		
	OR	95% CI	*p*-value	OR	95% CI	*p*-value
**Multiivariable model**						
Preoperative Global Quality of Life (QL)	0.94	0.93–0.94	< *0.001*	0.88	0.87–0.89	< *0.001*
Fulfilled trifecta criteria at 24 months after surgery	1.91	1.59–2.29	< *0.001*	1.61	1.27–2.04	< *0.001*
CAPRA-S risk group (high risk vs. low risk [REF])	0.60	0.46–0.78	< *0.001*	0.67	0.48–0.94	*0.02*
Surgery complications (CDC ≥ 3b vs. 0–3a[REF])	0.49	0.24–0.99	*0.048*	0.94	0.42–2.10	0.9
**Univariable model**						
Preoperative Global Quality of Life (QL)	0.94	0.93–0.94	< *0.001*	0.89	0.88–0.90	< *0.001*
Fulfilled trifecta criteria at 24 months after surgery	1.76	1.50–2.05	< *0.001*	1.53	1.25–1.87	< *0.001*
CAPRA-S risk group (high risk vs. low risk [REF])	0.63	0.51–0.79	< *0.001*	0.68	0.52–0.91	*0.008*
Surgery complications (CDC ≥ 3b vs. 0–3a[REF])	0.64	0.38–1.07	0.09	1.39	0.79–2.46	0.3

Significant values are in italics.

*CAPRA-S* the postsurgical Cancer of the Prostate Risk Assessment score, *CDC* Clavien–Dindo classification, *CI* confidence interval, *OR* odds ratio.

LRM for prediction of improved Summary Score at 24 months after RARP yielded similar results. However, CDC did not reach independent predictor status for aforementioned scales (OR 0.94, 95% CI 0.42–2.10; p = 0.9).

Finally, LRM for prediction of at least stable or improved QL (or Summary Score) at 24 months, which represents a broader definition of a desirable outcome than just improvement, yielded comparable results (Supplemental Table 1).

## Discussion

To date, postoperative QoL metrics in combination with MCID are still rarely reported in context of specific functional or oncological profiles of PCa patients, which were treated with RARP. Interpretability of QoL change is furthermore limited due averaged data, e.g. observed mean EORTC QLQ-C30 values before and after surgery. This requires rectification since QoL metrics serve as important patient counselling tool before surgery, monitoring after surgery and benchmarking tool for comparison with other institutions^35,36^. Thus, we compared the impact of specific pathological PCa profiles and of the widely adopted pentafecta criteria on key measurements of the EORTC QLQ-C30.

Our study revealed important findings. First, median preoperative QL and Summary Score values were virtually identical between CAPRA-S groups, as well as pentafecta vs. non-pentafecta groups. Such preoperative balance indicates validity of our MCID findings, since MCIDs are calculated as the difference between preoperative vs. postoperative values. Moreover, our preoperative values are highly consistent with randomized trials and indicate a healthy baseline functioning in the majority of our RARP patients and adequate patient selection^17–19^.

Second, despite balanced and healthy baseline values, we are the first to report substantial proportions of RARP patients who improve upon their preoperative global QoL (47%) and Summary Score (20%). Reassuringly, depending on scale, the vast majority of pentafecta patients, 77% (QL) to 80% (Summary Score) do not show any deterioration after RARP, but either remain stable or improve further.

Third of those pentafecta patients reporting deterioration, 8–10%, about a third, are only classified as minor. It is important to note, that deterioration might occur in a specific scale but be absent in another. This is evidenced by the mean change of QL, which is positive in CAPRA-S LR and pentafecta patients and negative in the Summary Score even in the same patients. This is consistent with the general observation of perceived deterioration in cancer patients despite successful treatment and favourable prognosis^37^.

Fourth, this phenomenon of discrepant perception in scales is further substantiated by multivariable analyses. Despite congruent patterns of the respective impact of CAPRA-S LR and pentafecta profiles on individual QL and Summary Score scales, LRM clearly demonstrated that CAPRA-S risk profiles are independent of the pentafecta criteria. For example, an individual with CAPRA-S high-risk profile and simultaneous pentafecta criteria might perceive certain scales as deteriorated due to greater weight on PCa risk than outcomes. In consequence, pathology and functional outcomes, should be considered in patients counselling.

It is important to note that a high-risk CAPRA-S score strongly correlates with adjuvant/salvage androgen deprivation therapy and/or radiotherapy, which is expected^12,30^. Accordingly, in context of QoL, an increasing CAPRA-S should also be interpreted as higher risk of biochemical recurrence and administration of additional therapy after RARP that potentially further contributes to a negative impact on QoL^1,18,32,38–40^.

With regard to functional outcomes, erectile aid such as phosphodiesterase type-5 inhibitors or intraurethral/intracavernosal application of alprostadil and urinary continence treatments such as male sling procedures or artificial urinary sphincter implantation ideally promote recovery of erectile function or urinary continence, respectively. These might translate to stable or improved QoL. This notion is substantiated by about 44% of preoperatively potent patients in our pentafecta group, who used erectile aids at time of erectile function recovery.

Finally, it is also important to point out that CAPRA-S LR vs. HR represents a distinct contrast, whereas pentafecta vs. non-pentafecta does not. Specifically, the difference is highly variable and might only contain minor differences compared to the very strict concept of pentafecta. However, this notion strengthens our findings of significant differences between pentafecta vs. non-pentafecta effects on QoL.

Our study has limitations. First, despite regular adoption of EORTC QLQ-C30 in randomized cancer trials, most MCIDs are not validated yet for PCa. We utilized the widely accepted MCID definition by Osoba et al.^29^ Although it was based on patients with breast and small-cell lung cancer, several subsequent series confirmed 10 point changes or more as being clinically significant after collating results from various studies and various cancer sites including PCa^41,42^. Our approach of MCIDs enables superior interpretation compared to simply reporting mean value as the majority of preceding series.

Second, we are also the first to report the validated EORTC QLQ-C30 Summary Score for RARP patients to complement global QoL^11,26–28^. Specifically, the Summary Score was validated as prognostic factor for survival^11^. Moreover, we aimed to improve interpretability and robustness of the scales/items in concatenated fashion as opposed to presenting each individual scales/items. Since the Summary Score represents a mean of aforementioned components, MCIDs could be applied accordingly. However, MCIDs are not validated yet for the Summary Score. Nonetheless, the direction and extent of change is transparently demonstrated in our study.

Third, since pentafecta outcomes are composed of time-to-event variables it is mandatory to focus on a specific time point after RARP, e.g. 24 months, for purpose of examining the relationship between the pentafecta outcome and QoL. This represents an inherent flaw of the pentafecta concept. For example, longer post-RARP follow-up will be associated with higher oncological failure rates that lead to pentafecta exclusion vs. increased recovery rates that lead to pentafecta inclusion. At 24 months post-RARP, QoL is likely more influenced by functional recovery than BCR. Conversely, post-RARP QoL might further improve or deteriorate after 24 months, since previous series indicate that QoL values stabilize after up to 6 years in absence of further interventions^20^.

Finally, it is important to note that surgical expertise is essential to achieve favorable combined outcomes and that different learning curves are needed for specific outcomes^43,44^. Specifically, previous series reported a shorter learning curve for achieving satisfactory outcomes for continence, followed by a longer curve for potency and then for surgical margins^45^. This is evidenced by a significantly higher surgical expertise in the pentafecta group (median 767, IQR 174–212 vs. 552, IQR 140–1750; p < 0.001). In the same context, generalizability of our findings are limited since our department represents a specialized prostate cancer center with highly experienced robotic surgeons.

## Conclusions

We confirm that favourable pathology, oncological and functional outcomes independently confer not only stability, but even positive change on specific QoL measurements in the vast majority of RARP patients. However, optimal outcomes after treatment do not guarantee best perceived QoL, since up to one third of patients might experience deterioration. This warrants further investigation of how to capture the underlying cause and how to finally address and potentially solve these perceived negative effects of PCa despite successful RARP.

## Supplementary Information

Supplementary Table 1.

## Data Availability

Full availability.

## Abbreviations

BCR: Biochemical recurrence
CAPRA-S: Postsurgical Cancer of the Prostate Risk Assessment score
CDC: Clavien–Dindo classification
EORTC QLQ-C30: European Organisation for Research and Treatment of Cancer Quality of Life Questionnaire Core 30
HR: High risk
IIEF-5: International Index of Erectile Function
LR: Low risk
LRM: Multivariable logistic regression analyses
MCID: Minimal clinical improvement difference
PCa: Prostate cancer
PROM: Patient-reported outcome measurement
PSA: Prostate specific antigen
RARP: Robot-assisted radical prostatectomy
RP: Radical prostatectomy
SD: Standard deviation
QL: Global quality of life
QoL: Quality of life

## Code availability

Full availability.
