# Supplementary material for: Two-year quality of life after robot-assisted radical prostatectomy according to pentafecta criteria and cancer of the prostate risk assessment (CAPRA-S)
Source: Sci Rep. 2022 Jan 7;12:244. doi: 10.1038/s41598-021-04289-2 (PMC8742105; doi:10.1038/s41598-021-04289-2)
Supplement: Supplementary file 1 — Supplementary Table 1. [file 41598_2021_4289_MOESM1_ESM.pdf]

Supplemental Table 1: Multi- and univariable logistic regression model for prediction of at least stable or improved Global Quality of Life (QL) and EORTC QLQ-C30 Summary Score at 24 months after robot-assisted radical prostatectomy

	Global Quality of Life (QL) no deterioration			EORTC QLQ-C30 Summary Score no deterioration		
**Value**	**OR**	**95% CI**	***p-value***	**OR**	**95% CI**	***p-value***
**Multiivariable model**						
Preoperative Global Quality of Life (QL)	0.97	0.96 - 0.97	*<0.001*	1.00	0.99 - 1.01	*0.4*
Fulfilled trifecta criteria at 24 months after surgery	1.90	1.60 - 2.26	*<0.001*	2.13	1.79 - 2.53	*<0.001*
CAPRA-S risk group (high risk vs. low risk [REF])	0.58	0.45 - 0.73	*<0.001*	0.74	0.59 - 0.94	*0.02*
Surgery complications (CDC ≥3b vs. 0-3a[REF])	0.31	0.17 - 0.56	*<0.001*	0.61	0.34 - 1.10	*0.1*
**Univariable model**						
Preoperative Global Quality of Life (QL)	0.97	0.96 - 0.97	*<0.001*	1.00	1.00 - 1.01	*0.3*
Fulfilled trifecta criteria at 24 months after surgery	1.95	1.66 - 2.30	*<0.001*	2.24	1.89 - 2.65	*<0.001*
CAPRA-S risk group (high risk vs. low risk [REF])	0.51	0.41 - 0.64	*<0.001*	0.61	0.49 - 0.77	*<0.001*
Surgery complications (CDC ≥3b vs. 0-3a[REF])	0.39	0.24 - 0.63	*<0.001*	0.74	0.44 - 1.22	*0.2*

CAPRA-S - the postsurgical Cancer of the Prostate Risk Assessment score, CDC - Clavien-Dindo classification, CI - confidence interval, OR - odds ratio
